# Enhancement of Cell-Based Therapeutic Angiogenesis Using a Novel Type of Injectable Scaffolds of Hydroxyapatite-Polymer Nanocomposite Microspheres

**DOI:** 10.1371/journal.pone.0035199

**Published:** 2012-04-18

**Authors:** Yohei Mima, Shinya Fukumoto, Hidenori Koyama, Masahiro Okada, Shinji Tanaka, Tetsuo Shoji, Masanori Emoto, Tsutomu Furuzono, Yoshiki Nishizawa, Masaaki Inaba

**Affiliations:** 1 Department of Metabolism, Endocrinology, and Molecular Medicine, Osaka City University Graduate School of Medicine, Osaka, Japan; 2 Department of Biomedical Engineering, School of Biology-Oriented Science and Technology, Kinki University, Wakayama, Japan; University of Illinois at Chicago, United States of America

## Abstract

**Background:**

Clinical trials demonstrate the effectiveness of cell-based therapeutic angiogenesis in patients with severe ischemic diseases; however, their success remains limited. Maintaining transplanted cells in place are expected to augment the cell-based therapeutic angiogenesis. We have reported that nano-hydroxyapatite (HAp) coating on medical devices shows marked cell adhesiveness. Using this nanotechnology, HAp-coated poly(l-lactic acid) (PLLA) microspheres, named nano-scaffold (NS), were generated as a non-biological, biodegradable and injectable cell scaffold. We investigate the effectiveness of NS on cell-based therapeutic angiogenesis.

**Methods and Results:**

Bone marrow mononuclear cells (BMNC) and NS or control PLLA microspheres (LA) were intramuscularly co-implanted into mice ischemic hindlimbs. When BMNC derived from enhanced green fluorescent protein (EGFP)-transgenic mice were injected into ischemic muscle, the muscle GFP level in NS+BMNC group was approximate fivefold higher than that in BMNC or LA+BMNC groups seven days after operation. Kaplan-Meier analysis demonstrated that NS+BMNC markedly prevented hindlimb necrosis (*P*<0.05 vs. BMNC or LA+BMNC). NS+BMNC revealed much higher induction of angiogenesis in ischemic tissues and collateral blood flow confirmed by three-dimensional computed tomography angiography than those of BMNC or LA+BMNC groups. NS-enhanced therapeutic angiogenesis and arteriogenesis showed good correlations with increased intramuscular levels of vascular endothelial growth factor and fibroblast growth factor-2. NS co-implantation also prevented apoptotic cell death of transplanted cells, resulting in prolonged cell retention.

**Conclusion:**

A novel and feasible injectable cell scaffold potentiates cell-based therapeutic angiogenesis, which could be extremely useful for the treatment of severe ischemic disorders.

## Introduction

Severe ischemic vascular disease is caused by atherosclerotic occlusion of the arteries supplying blood to the myocardium and limbs. We and other groups have reported the clinical efficacy and safety of therapeutic angiogenesis by cell transplantation as an optional treatment for these diseases [1]–[4], although the effectiveness of this approach is still limited [5]. Various cell types, including autologous bone marrow mononuclear cells (BMNCs), stem cells, endothelial progenitor cells (EPCs), and peripheral blood mononuclear cells (PBMNCs), have been implanted to induce neovascularization for the treatment of ischemic heart disease and peripheral arterial disease [1]–[4], [6]. With few exceptions, the administration route is injection into the ischemic tissue. Various angiogenic growth factors derived from implanted cells are key mediators of therapeutic angiogenesis [3], [7], [8]. However, about 70%–90% of the transplanted cells were estimated to disappear from the injection site within 1 week after transplantation [9]–[11]. Therefore, the efficacy of the cell-based therapeutic angiogenesis could be dependent on the retention, survival, and engraftment of implanted cells in ischemic tissue after implantation.

In order to enhance the retention, survival, and engraftment of implanted cells, several approaches have been reported including genetic manipulation of donor cells (e.g., overexpression of antiapoptotic genes) [1], [6], [7] and cell delivery strategies using matrix scaffolds [12]–[14]. These strategies were reported to successfully promote long-term cell survival and capillary formation, resulting in functional recovery of perfusion in ischemic organs. However, the cumbersome procedures and techniques required may be an obstacle to the treatment of large numbers of cells in clinical use. Therefore, simple, safe and effective novel approaches to retain transplanted cells in place are urgently required.

Hydroxyapatite (HAp) is the main mineral component of bones and teeth, and artificially synthesised HAp has been used extensively as a biomaterial because of its excellent adhesive properties, not only to hard and soft tissues but also to cells of various types [15]. However, due to the mechanical weakness and brittleness of HAp ceramics, they have been confined to applications with low mechanical stress. To overcome its mechanical weakness, we recently developed a high-dispersed and crystallised nano-scale HAp coating for flexible polymer substrates [16]–[18]. The novel composite retained most of the mechanical properties of the substrate [16] and showed improved cell adhesion and reduced inflammatory reaction due to the surface HAp nanocrystals [17]–[20]. Poly(l-lactic acid) (PLLA) microspheres were used as a polymer substrate, to generate the biodegradable and injectable cell scaffold. PLLA is one of the most promising biodegradable polymers and has been used in the fields of orthopaedic and reconstructive surgery and tissue engineering [21] as it is not necessary to remove the material after healing. However, PLLA has the disadvantage of low cell adhesion due to its hydrophobic surface [22]. Our HAp nanocrystal coating is expected to overcome the disadvantages of PLLA microspheres [20]. Thus, nano-scale HAp-coated PLLA microspheres, named nano-scaffold (NS), were generated using our nanotechnology as a non-biological, biodegradable and injectable type of cell scaffold. In this study, we investigated the ability of NS to enhance cell-based therapeutic angiogenesis by retention of implanted cells.

## Methods

### Animals

BALB/cAJcl mice were purchased from Clea Japan, Inc. (Tokyo, Japan). Enhanced green fluorescent protein (EGFP)-transgenic C57/BL6-Tg (CAG-EGFP) mice and wild-type C57BL/6NCrSlc mice were purchased from Japan SLC, Inc. (Hamamatsu, Japan). The procedures used in this study were approved by the Animal Care and Use Committee of Osaka City University Graduate School of Medicine, Osaka, Japan (approval number, 09023).

### Materials

The PLLA microspheres (PLA-Particles), PLLA microspheres containing rhodamine B (PLA-Particles-redF) and PLLA microspheres containing magnetite (PLA-Particles-M) were purchased from Micromod Partikeltechnologie (Rostock, Germany).

Rat anti-CD31 antibody (clone: MEC13.3) was obtained from BD Pharmingen (San Diego, CA). The avidin-biotin complex (ABC) kit and the 3,3′-diaminobenzidine (DAB) substrate kit were purchased from Vector Laboratories (Burlingame, CA). Rat monoclonal anti-vascular endothelial growth factor (VEGF) antibody (clone: RM0009-2G02) was purchased from Angio-Proteomie (Boston, MA). Rabbit polyclonal anti-fibroblast growth factor-2 (FGF-2) antibody was obtained from Abcam (Cambridge, UK). Alexa Fluor 594-conjugated goat anti-rat IgG antibody and Alexa Fluor 594-conjugated chicken anti-rabbit IgG antibody were purchased from Invitrogen Life Technologies (Carlsbad, CA). 4′,6-Diamino-2-phenylindole dihydrochloride (DAPI) was obtained from Pierce Biotechnology (Rockford, IL). Enzyme-linked immunosorbent assay (ELISA) kits for VEGF, FGF-2, interleukin 1-β (IL-1β), monocyte chemotactic protein-1 (MCP-1) and stromal cell-derived factor-1 (SDF-1) were purchased from R&D Systems (Minneapolis, Minn). The GFP ELISA kit was obtained from Cell Biolabs (San Diego, CA). Barium sulphate was purchased from Sakai Chemical Industry (Osaka, Japan) and used as contrast material for three-dimensional computed tomography (3D-CT) angiography.

### Fabrication of HAp nanocrystals and injectable cell scaffold

The dispersed HAp nanocrystals with an average diameter of 50 nm and rod-like shape were prepared with a wet chemical process and used after calcination with an antisintering agent—poly(acrylic acid, calcium salt)—at 800°C for 1 hour, as described in our previous reports [23]. HAp nanocrystal-coated PLLA microspheres were fabricated as described [24]. Briefly, the PLLA microspheres (0.1 g) were treated with alkali (pH 11.0; adjusted with 25% ammonia solution) for 1 h at room temperature to introduce carboxyl groups onto the surfaces, washed with water, and then dried under reduced pressure. The alkali-treated and dried microspheres were washed with ethanol and immersed in a 1.0% HAp ethanol dispersion for 1 h at room temperature with stirring. The HAp-coated PLLA was washed five times with ethanol by sonication for 3 min, dried under reduced pressure, and used as NS. Bare PLLA microspheres (LA) were used as controls. The NS and LA were characterised in terms of size, particle size distribution and morphology using scanning electron microscopy.

### BMNC isolation

Isolation of BMNCs was performed as described [25]. Briefly, BMNCs were harvested from 8-week-old male mice [BALB/cAJcl or C57/BL6-Tg (CAG-EGFP)] by washing of the tibiae and femora with serum-free Dulbecco's Modified Eagle's Medium (DMEM) and separated using Ficoll-Paque Plus (GE Healthcare AB, Sweden).

### BMNC adhesion on microspheres *in vitro* and scanning electron microscopy observation

Murine BMNCs (5×10^6^ cells) were incubated with each microsphere preparation (3,000 particles) at 37°C for 8 h. After incubation, microspheres were harvested using a cell strainer (35-µm nylon mesh; BD Falcon, Japan). The samples were fixed with 2.5% glutaraldehyde for 1 h and dehydrated with aqueous ethanol (30%, 50%, 70%, 90%, 99%, 100%) media and 100% *n*-butanol for 15 min in each step. They were subsequently lyophilized and observed under scanning electron microscopy (SEM) (JSM-6301F; JEOL Ltd., Tokyo, Japan) operated at 5 kV.

### BMNC transplantation in hind limb ischemia model

Eight-week-old male mice (BALB/cAJcl or C57BL/6NCrSlc) were used for the hind limb ischemia model. Unilateral hind limb ischemia was induced by resection of the left femoral arteries, veins and side branches under anaesthesia with sodium pentobarbital (50 µg/g) injected intraperitoneally (i.p.) as described [26]. BMNCs (5×10^6^ cells) were suspended in 100 µL of serum-free DMEM with or without 1.5 mg (3,000 particles) of NS or LA, and incubated at 37°C for 3 h. Immediately after operative resection of the artery and vein, 100 µL of the suspension including 5×10^6^ cells and/or 3,000 particles of each microsphere preparation was injected intramuscularly into the ischemic thigh muscle at 4 sites (25 µL/site) using a 22-gauge needle as described elsewhere [27]. Ischemic operation, cell transplantation and evaluation of limb necrosis were performed by separate operators in a blinded manner.

### Measurement of GFP and proangiogenic factors in ischemic tissue

We collected whole thigh muscle tissues 3, 7 and 14 days after induction of limb ischemia. The tissue lysate was prepared using a modification of the technique described by Abbott *et al.* [28]. Briefly, tissue extracts from thigh muscle were prepared by homogenization and lysis with 50 mM Tris-HCl, 5 mM EDTA, 250 mM NaCl, 50 mM NaF, 0.5 mM Na_3_VO_4_, 10 mM β-GP, 0.5% NP-40 and protease inhibitor cocktail (Nacalai Tesque, Inc. Japan). The homogenate was centrifuged for 5 min at 10,000×*g*. The clear supernatant was collected and used for quantification of GFP, proangiogenic factors (VEGF, FGF-2, MCP-1, SDF-1 and IL-1β) and total protein content. Total protein content of each sample was determined by bicinchoninic protein assay. Proangiogenic factors and GFP were quantified using the respective ELISA kits in accordance with the manufacturer's instructions.

### Immunohistochemistry and analysis of vessel numbers

Thigh tissue sections were prepared using a modification of the technique described by Clausen *et al.* [29]. Briefly, mice were anaesthetized with pentobarbital (i.p.) and perfused through the left ventricle using 20 ml of saline followed by 100 ml of cold 4% paraformaldehyde (PFA) in phosphate buffer (PB). The thigh tissue was post-fixed in 4% PFA for 2 h. The tissue was then embedded in paraffin or immersed in 20% sucrose in PB overnight followed by freezing in OCT compound (Sakura Finetek, Co. Japan). Paraffin-embedded sections were used for immunohistochemical staining of CD31 as described [30]. Following deparaffinization and rehydration of the sections, endogenous peroxidase activity was blocked using 0.3% hydrogen peroxide before application of blocking serum. Sections were then incubated with anti-CD31 antibody. Immunohistochemical staining was performed by the avidin-biotin complex method (ABC Kit). Colour was developed with DAB. Sections were counterstained with hematoxylin (Sigma Diagnostics, MO). CD31-positive capillary numbers were counted in 4 fields of the injection site. The injection site landmarks were the microspheres (NS or LA). The frozen sections were incubated with anti-mouse VEGF antibody or anti-mouse FGF-2 antibody, followed by incubation with fluorescent secondary antibodies and counterstaining with DAPI. Immunofluorescence was observed using a fluorescence microscope (BZ-8000; Keyence, Osaka, Japan).

### CT analysis, 3D-CT angiography, and vascular volume measurement

We used PLLA microspheres containing magnetite (PLA-Particles-M) as control LA and the core of NS to be detected by X-ray CT scan. Angiography was performed using the technique described by Zhuang *et al.* [31] with slight modifications. Briefly, BALB/cAJcl mice were anaesthetised with sodium pentobarbital (50 µg/g, i.p.) and injected with 5 µl/g barium sulphate suspension (90 w/v%) directly into the left ventricle. CT images were obtained using a micro-CT scanner (La Theta LCT200; Aloka, Tokyo, Japan) according to the manufacturer's protocol. The 3D data were constructed from sliced CT images by summing those images along the Z-axis, and vascular volume analysis was performed with an image analyzer (VGStudio MAX software; Volume Graphics, Heidelberg, Germany) according to the manufacturer's protocol.

### Terminal deoxynucleotidyl transferase-mediated biotin-16-dUTP nick-end labelling (TUNEL) Assay

Apoptotic transplanted BMNCs and host cells were evaluated 10 days after limb ischemia by TUNEL assay in frozen sections with an *In Situ* Cell Death Detection Kit, TMR Red (Roche Laboratories) in accordance with the instructions provided by the manufacturer.

### Statistical analysis

Data are presented as means (SD). Statistical significance was evaluated by ANOVA and Scheffé's test for comparison and contrast between multiple groups. Plots of the estimated limb survival ratio after the operation were constructed by the Kaplan-Meier method and were compared using the log-rank test. In all analyses, *P*<0.05 was taken to indicate significance.

Additional descriptions of experimental procedures can be found in Methods S1.

## Results

### Characteristic of nano-scaffold (NS) with enhanced adhesiveness of bone marrow mononuclear cells (BMNCs) *in vitro*

**Figure 1:**
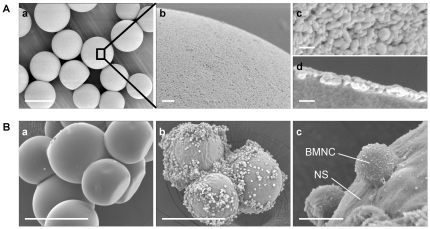
SEM image of NS (A) and marked cell adhesiveness to NS *in vitro* (B). (A) NS are microspheres approximately 100 µm in diameter (a). The NS surface uniformly coated with nano-scale hydroxyapatite (HAp) crystals was observed at different magnifications (low and high magnification in b and c, respectively). SEM image of an NS cross-section indicating a single layer of nano-scale HAp particles on the NS surface (d). (B) Murine BMNCs were incubated with LA (a) or NS (b, c) at 37°C for 8 h. Large numbers of BMNCs adhered to NS (b, c) but not to LA (a). Scale bars: 100 µm (A-a, B-a, B-b), 5 µm (B-c), 1 µm (A-b), 100 nm (A-c, A-d). Abbreviations: SEM, scanning electron microscopy; NS, nano-scaffolds; LA, unmodified PLLA microspheres; BMNCs, bone marrow mononuclear cells.

NS are microspheres approximately 100 µm in diameter (Fig. 1A-a), the surfaces of which are coated with a monolayer of HAp nanoparticles 50 nm in diameter (Fig. 1A-b, -c, -d). To assess the cell adhesiveness of NS, SEM was performed after incubation of NS and bare PLLA microspheres (LA) as controls with murine BMNCs at 37°C for 8 h *in vitro*. The number of cells adhering to NS was much greater than that to LA (Fig. 1B-a and b). High-magnification SEM images showed active cell adhesion to NS (Fig. 1B-c).

### NS prolonged localization of implanted BMNCs in ischemic tissue

**Figure 2:**
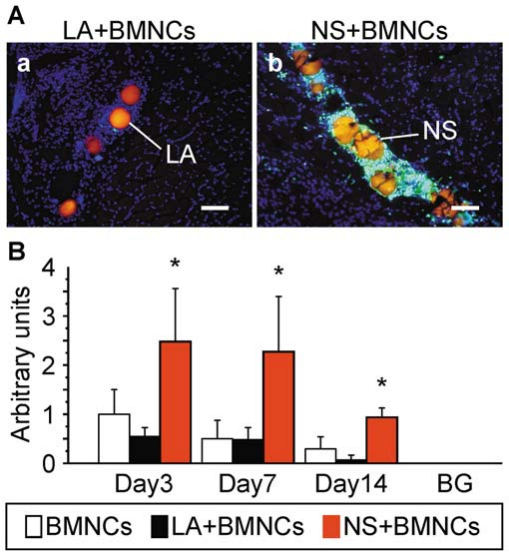
Prolonged localization of implanted BMNCs in ischemic tissues by NS. (A) Colocalization of BMNCs with NS and LA *in vivo*. Murine BMNCs derived from EGFP-transgenic mice were transplanted together with LA or NS into the thighs in the hind limb ischemic model. Cores of NS and LA containing rhodamine B (orange) were used to indicate localisation of the injected microspheres in ischemic tissues. Tissue sections 7 days after transplantation of LA+BMNCs (a) or NS+BMNCs (b) were counterstained with DAPI (blue), and merged images of DAPI, GFP and rhodamine B are shown. BMNCs (green) were observed as densely clustered around NS (b) but not LA (a). Scale bars: 100 µm. (B) Quantitative evaluation of implanted cells existing in ischemic tissues. Quantitative analysis of intramuscular GFP was performed 3, 7 and 14 days after transplantation. BMNCs were derived from EGFP-transgenic mice. BMNCs were transplanted alone or together with LA or NS into ischemic thigh muscles. Intramuscular GFP values of whole thigh muscles were corrected for total protein and expressed in arbitrary units (n = 6 in each group). *P<0.05 for the NS+BMNCs group compared to the BMNCs alone and LA+BMNCs groups. GFP concentration in normal murine muscle was measured as background (BG). Abbreviations: NS, nano-scaffolds; LA, unmodified PLLA microspheres; BMNCs, bone marrow mononuclear cells.

To determine the colocalization of implanted cells with injected microspheres, BMNCs from EGFP-transgenic mice and rhodamine B-containing PLLA microspheres (orange) as a scaffold core or control microspheres were implanted into the ischemic hind limbs of C57BL/6NCrSlc mice (Fig. 2A). Few implanted BMNCs were observed around LA (Fig. 2A-a), while markedly larger numbers of cells were seen with NS (Fig. 2A-b) in ischemic thigh tissue 7 days after transplantation. Intramuscular levels of GFP derived from transplanted BMNCs were consistently and significantly higher in the group injected with NS than that injected with LA or BMNCs alone at 3, 7 and 14 days after implantation, while GFP levels were not significantly different between BMNCs alone and LA+BMNCs groups (Fig. 2B).

### Co-implantation of NSs enhances limb salvage by BMC implantation

**Figure 3:**
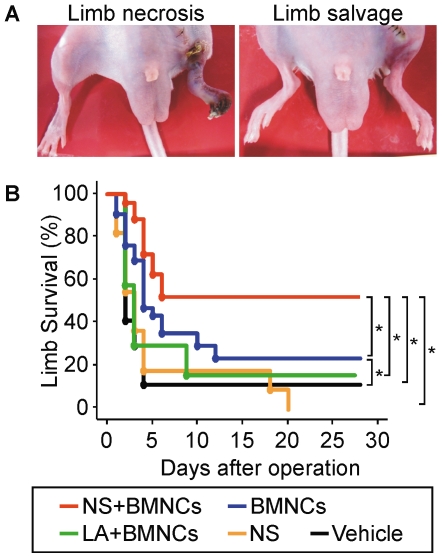
NS enhance limb salvage by BMNC transplantation. (A) Representative photographs of mice with limb necrosis (left) and limb salvage (right). Limb necrosis was evaluated every day after the ischemic operation in a blinded manner. (B) The survival curve for limb necrosis of hind limb ischemic mice (BALB/cAJcl) after ischemic induction and simultaneous intramuscular implantation of NS+BMNCs (*n* = 32), BMNCs alone (*n* = 33), LA+BMNCs (*n* = 7), NS alone (*n* = 11) or vehicle alone (*n* = 17). The curve was obtained using the Kaplan-Mayer method and the difference between the 2 groups was compared using the log-rank test. *: *P*<0.05. Abbreviations: NS, nano-scaffolds; LA, unmodified PLLA microspheres; BMNCs, bone marrow mononuclear cells.

Hind limb ischemia in BALB/c mice was used as an intractable ischemia model as these mice show little spontaneous collateral vessel formation in response to ischemia with ischemic hind limb necrosis [1] (Fig. 3A). The limb survival ratios after the operation in each group were compared using Kaplan-Meier analysis and log-rank statistics (Fig. 3B). In this limb ischemic model, approximately 90% of mice treated with vehicle alone developed hind limb necrosis within 5 days after the operation. Injection of NS alone did not improve limb survival, while BMNC implantation slightly but significantly improved the limb survival ratio to about 20%. Implantation of BMNCs with NS (NS+BMNCs group) markedly improved limb survival compared with implantation of BMNCs alone, while co-injection of LA did not.

### Co-implantation of NSs and BMNCs enhances angiogenesis and arteriogenesis

**Figure 4:**
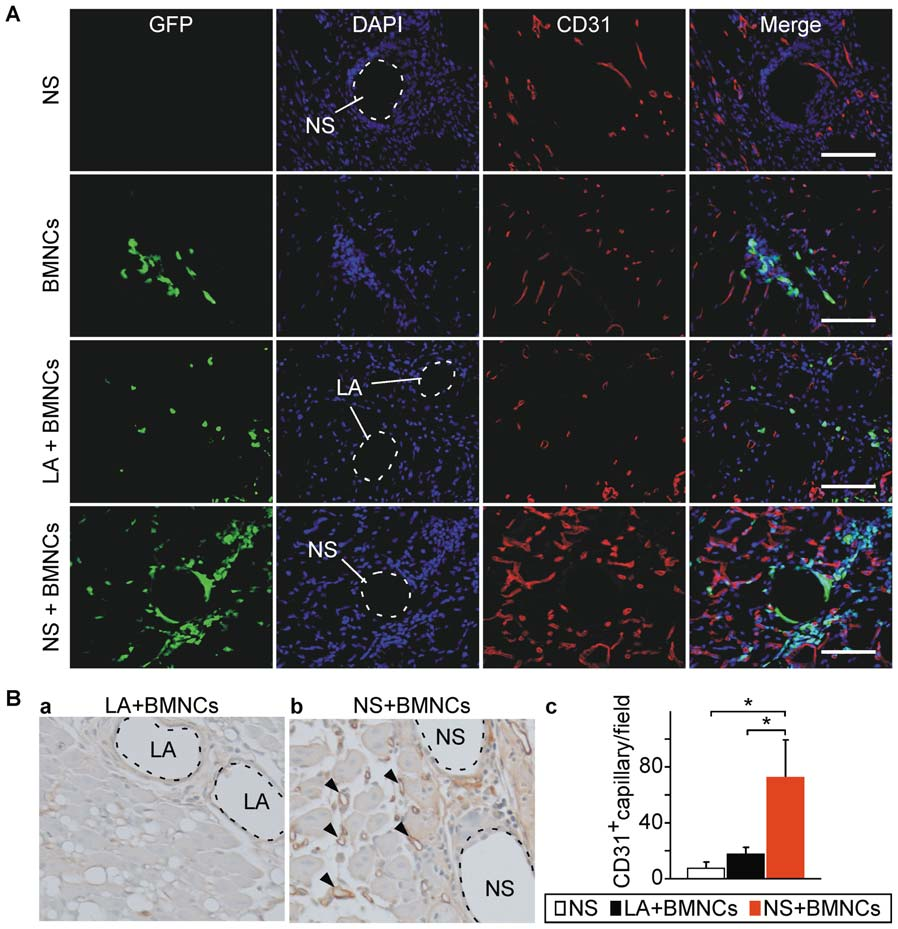
Angiogenesis in hind limb ischemic model. (A) Tissue sections from hind limb ischemic mice (C57BL/6NCrSlc) 7 days after transplantation of NS, BMNCs, LA+BMNCs or NS+BMNCs were immunofluorescently stained using anti-mouse CD31 antibody (red) and counterstained with DAPI (blue). BMNCs (green) were derived from EGFP-transgenic mice. The areas circled with dashed lines indicate the presence of NS or LA. Scale bars, 100 µm. (B) Quantitative evaluation of capillary density was performed by immunohistochemical staining using anti-mouse CD31 antibody in ischemic hind limbs of mice (BALB/cAJcl) 7 days after transplantation of NS, LA or NS+BMNCs. Typical staining of CD31-positive capillaries in high-power field in LA+BMNCs and NS+BMNCs groups are shown in (a) and (b), respectively. Arrowheads indicate representative CD31-positive capillaries. CD31-positive capillary numbers were counted in 4 low-power fields of the injection site, which had microspheres (LA or NS) as landmarks in each mouse (*n* = 3 in each group) (c). Data are shown as means (SD). *P<0.05 for the NS+BMNCs group compared to the NS and LA+BMNCs groups. Abbreviations: NS, nano-scaffolds; LA, unmodified PLLA microspheres; BMNCs, bone marrow mononuclear cells.

To determine whether NS enhances cell implantation-induced angiogenesis, capillary formation associated with implanted BMNCs derived from EGFP-transgenic mice was determined by immunostaining for the endothelial cell marker CD31 (Fig. 4A). The density of CD31-positive capillaries in mice implanted with NS alone (Fig. 4A) was the same as that in the group injected with vehicle alone (data not shown). In the LA+BMNCs group, most of the GFP-positive BMNCs disappeared from around the LA and were no longer colocalized with LA 7 days after transplantation (Fig. 4A), and capillary density around migrating BMNCs was somewhat increased. In contrast, capillary density was markedly increased when BMNCs were co-implanted with NS. Similar enhanced CD31-positive capillary formation in the NS+BMNCs group compared with the LA+BMNCs group was observed in another severe ischemia model in BALB/cAJcl mice (Fig. 4B-a, b, c). If BMNCs alone are implanted, there will be hardly any BMNCs in the implanted area after 7 days (Fig. 2B), thereby complicating the evaluation of capillary formation in the implanted area. Because BMNCs do not adhere to LA, the BMNCs implanted in the LA+BMNCs group will also migrate from the implanted area, and there will be hardly any BMNCs remaining in the implanted area after 7 days, similar to the BMNCs alone group (Fig. 2, 4A). For this reason, LA, which is evaluated as a target for comparison with an NS particle in this study, additionally serves as a marker of the BMNCs-implanted area.

**Figure 5:**
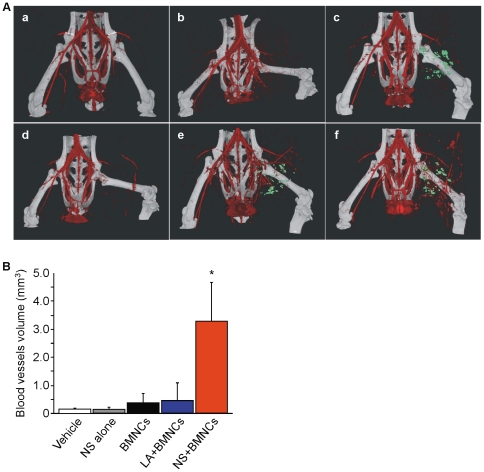
Collateral vessel formations in hind limb ischemic model. (A) 3D-CT angiography of mice (BALB/cAJcl) was performed immediately after the operation (a) and 7 days after implantation of vehicle alone (b), NS alone (c), BMNCs alone (d), LA+BMNCs (e) or NS+BMNCs (f). Representative 3D-CT angiograms are presented. PLLA microspheres containing magnetite (PLA-Particles-M) were used as core NS and LA to be detected by X-ray 3D-CT. Detected NS and LA by 3D-CT were visualised as green particles. (B) Quantitative volume analysis of collateral vessels in the ischemic area used the arterial phase 3D-CT angiogram data (n = 3 in each group). Data are shown as means (SD). *P<0.05 for the NS+BMNCs group compared to the vehicle, NS alone, BMNCs alone, and LA+BMNCs groups. Abbreviations: NS, nano-scaffolds; LA, unmodified PLLA microspheres; BMNCs, bone marrow mononuclear cells.

Next, we performed X-ray 3D-CT angiographic analysis to determine collateral vessel formation and blood flow. Blood flow of the left hind limb was confirmed to be completely disrupted just after femoral artery resection (Fig. 5A-a). As our micro-CT equipment has a spatial resolution of 48-µm, NS or LA with a diameter of 100 µm could be readily detected, and were visualised as light green particles on 3D-CT images. No collateral arteries had developed 7 days after the operation in the vehicle (Fig. 5A-b) or NS alone groups (Fig. 5A-c). Collateral arteries were somewhat enhanced in the BMNCs alone (Fig. 5A-d) and LA+BMNCs groups (Fig. 5A-e). Interestingly, collateral arteries were well developed around NS in the NS+BMNCs group (Fig. 5A-f). The vascular volume of the ischemic area was measured with 3D-CT angiogram data (Fig. 5B). For objective evaluation of the development of collateral arteries in the ischemic areas, we measured the vascular volume within the lower part of the thigh, ranging from the center of femur's major axis to the end of femur, based on the arterial phase angiographic data. Resembling the development of microvessels not more than 10 µm in diameter (Fig. 4), the collateral arteries that were detectable with 3D-CT also showed more marked increase in the NS+BMNCs group as compared to that in the BMNCs alone group and the LA+BMNCs group (Fig. 5B).

### Co-implantation of NSs augments local production of proangiogenic factors induced by BMNC implantation

**Figure 6:**
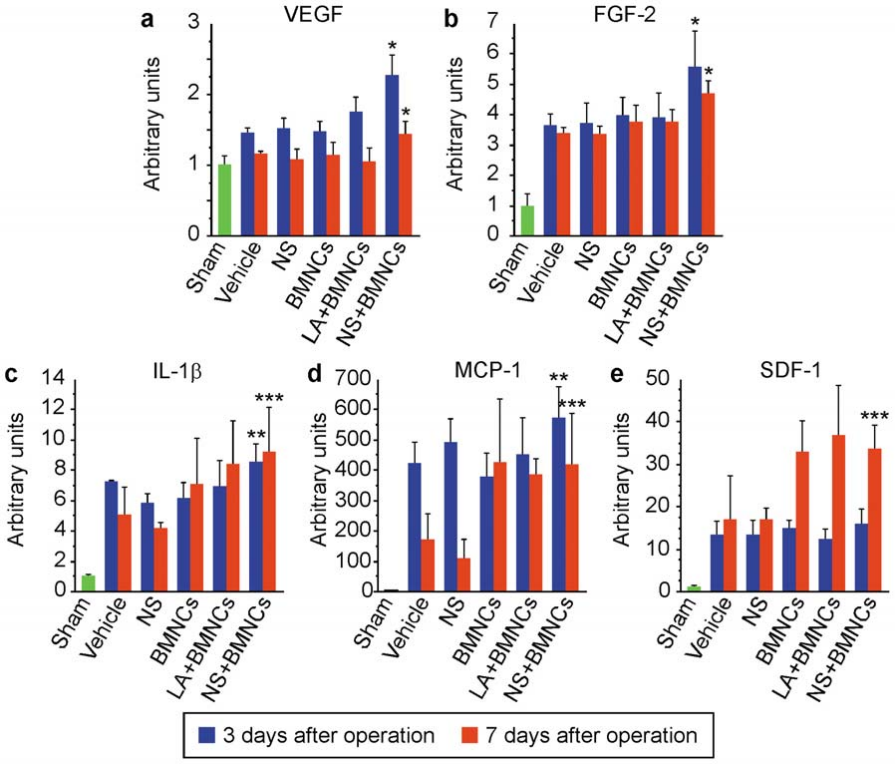
Expression of proangiogenic factors in ischemic hind limb muscles treated with transplantation. Intramuscular levels of proangiogenic factors in whole thigh muscles were quantified in hind limb ischemic model (BALB/cAJcl mice) 3 and 7 days after ischemic induction. Vehicle alone, NS alone, BMNCs alone, LA+BMNCs or NS+BMNCs were injected into ischemic thigh muscles simultaneously with ischemic induction. Intramuscular levels of proangiogenic factors were corrected for total protein and expressed in arbitrary units (n = 6 in each group). Data are shown as means (SD). *, *P*<0.05 for the NS+BMNCs group compared to the non-ischemic muscle (sham), vehicle, NS, BMNCs and LA+BMNCs groups. **, *P*<0.05 for the NS+BMNCs group compared to the non-ischemic muscle (sham), vehicle, NS and BMNCs groups. ***, *P*<0.05 for the NS+BMNCs group compared to the non-ischemic muscle (sham), vehicle and NS groups. Abbreviations: NS, nano-scaffolds; LA, unmodified PLLA microspheres; BMNCs, bone marrow mononuclear cells.

To investigate which proangiogenic factors are involved in enhanced therapeutic angiogenesis in mice treated with NS and BMNCs, candidate cytokines, including VEGF, FGF-2, IL-1β, MCP-1 and SDF-1 were measured by ELISA in ischemic hind limb muscles of the BALB/c mouse. Intramuscular levels of these proangiogenic factors were unaffected by vehicle injection alone (data not shown). Intramuscular levels of VEGF and FGF-2 in the NS+BMNCs group were significantly higher than those in the other groups 3 and 7 days after the operation (Fig. 6-a, -b). Levels of IL-1β and MCP-1 in the NS+BMNCs group were slightly but significantly higher than those in the BMNCs group but not those in the LA+BMNCs group 3 days after the operation (Fig. 6-c, -d). However, the levels of IL-1β and MCP-1 were not significantly different among BMNCs alone, LA+BMNCs, and NS+BMNCs groups 7 days after the operation (Fig. 6-c, -d). The level of SDF-1 in the NS+BMNC group was comparable to those in other groups with ischemia at 3 days after the operation. Although SDF-1 levels in the BMNCs alone, LA+BMNCs, and NS+BMNCs groups were higher 7 days after the operation than those in the other groups treated without BMNCs, there were no differences among the 3 groups treated with BMNCs (Fig. 6-e). Importantly, while VEGF levels were normalized even in the BMNCs and LA+BMNCs groups 7 days after ischemic operation, the NS+BMNCs group showed prolonged elevation of VEGF level (Fig. 6-a). This effect may explain enhanced recovery of blood flow in NS+BMNCs group, since long-term exposure to VEGF was reported to be necessary to produce stable microvasculature that is not resorbed after withdrawal of VEGF stimulation [3].

**Figure 7:**
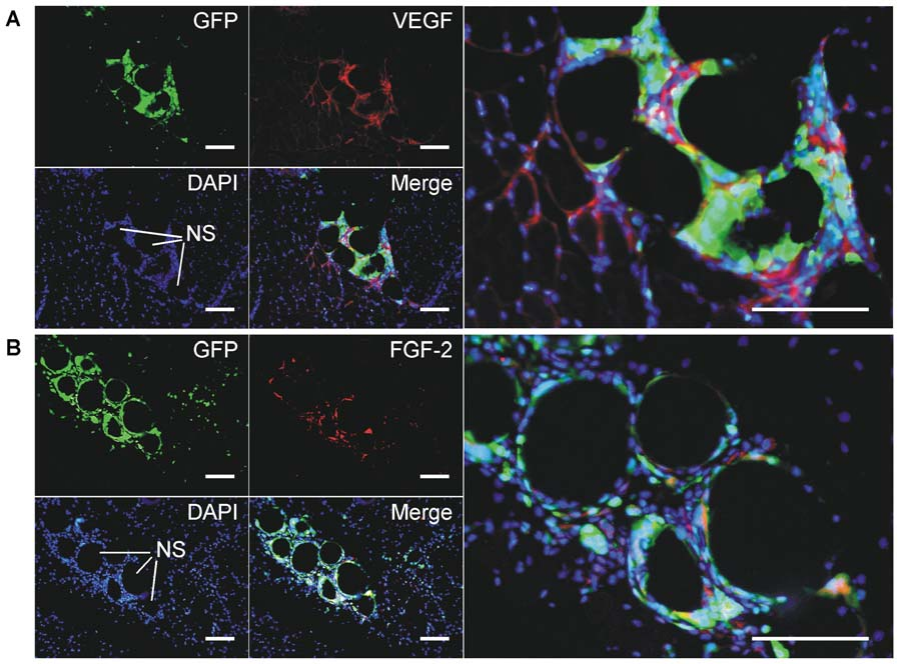
Colocalization of transplanted BMNCs and VEGF (A) or FGF-2 (B). Tissue sections from hind limb ischemic mice (C57BL/6NCrSlc) 7 days after transplantation of NS+BMNCs were immunofluorescently stained using anti-mouse VEGF antibody (A) or anti-mouse FGF-2 antibody (B) and DAPI. BMNCs were derived from EGFP-transgenic mice. Scale bars, 100 µm. Abbreviations: NS, nano-scaffolds; LA, unmodified PLLA microspheres; BMNCs, bone marrow mononuclear cells.

To further clarify the roles of proangiogenic factors, we investigated the localization of VEGF and FGF-2 expression in the ischemic muscle. VEGF expression was mostly co-localized with implanted GFP-positive BMNCs around NS, with some diffusing out of the clusters of BMNCs and NS (Fig. 7A). In contrast, although FGF-2-expressing cells were detected around and within the clusters, many did not express GFP (Fig. 7B), which could be explained by the previous finding that transplanted progenitor cells stimulate resident cells to produce additional secreted factors [8].

### Co-implantation of NSs prevents apoptosis of transplanted BMNCs

**Figure 8:**
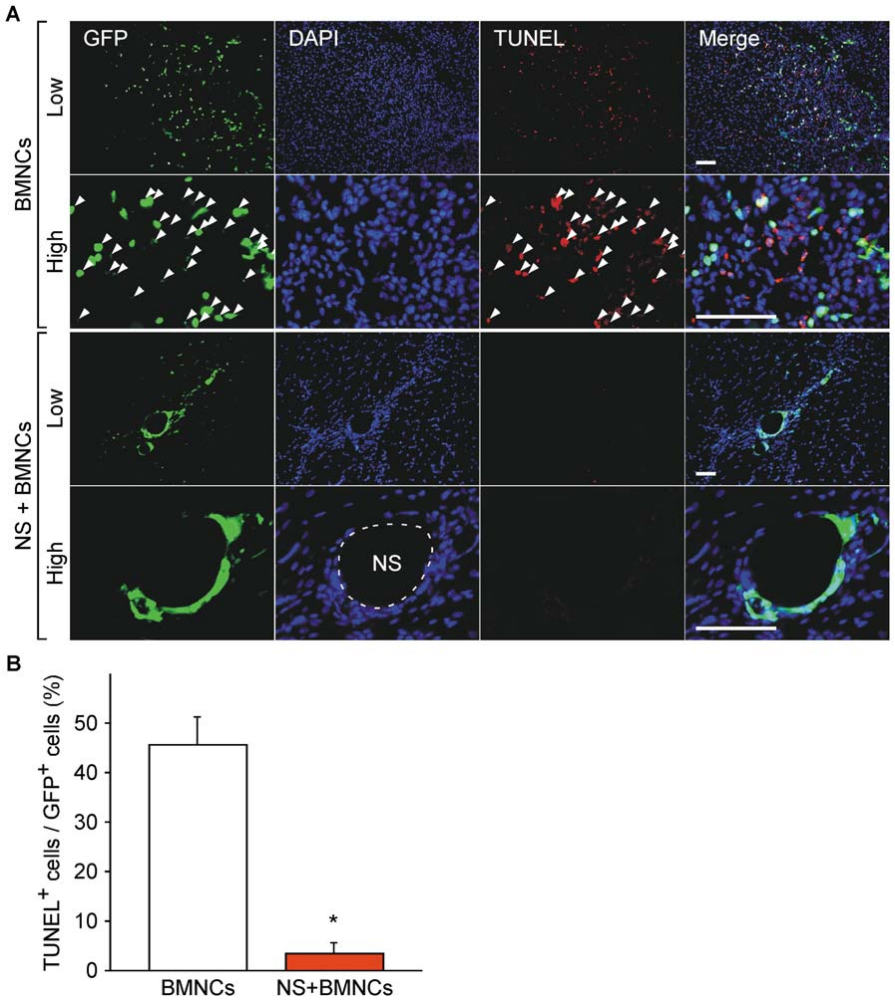
NS prevents apoptotic cell death of implanted cells. (A) BMNCs (green) were derived from EGFP-transgenic mice. Tissue sections from hind limb ischemic mice (C57BL/6NCrSlc) 10 days after transplantation were counterstained with DAPI (blue). Apoptotic nuclei were stained with tetramethylrhodamine (TMR) (red) by the TUNEL method. Arrowheads indicate cells double-positive for GFP and TUNEL. Scale bars, 100 µm. The upper panels of each group show low magnification (Low) and the lower panels show high magnification (High). (B) The percentage of TUNEL-positive cells out of GFP-positive ones was assessed in 4 low-power fields in each mouse (n = 3 in each group). Data are shown as means (SD). *P<0.05 for the NS+BMNCs group compared to the BMNCs group. Abbreviations: NS, nano-scaffolds; BMNCs, bone marrow mononuclear cells; DAPI, 4′,6-diamidino-2-phenylindole; TUNEL, terminal deoxynucleotidyl transferase-mediated dUTP-biotin nick-end labelling.

We investigated the possibility that co-implantation of NS prevents apoptotic cell death of BMNCs, and thus contributes to prolonged cell localization. Large numbers of transplanted BMNCs were positive for TUNEL 10 days after the operation when injected alone, suggesting apoptotic cell death (Fig. 8A-upper half, B). Some TUNEL-positive BMNCs did not necessarily express abundant GFP possibly due to the process of cell death. In marked contrast, TUNEL-positive BMNCs were hardly detected when cells were transplanted with NS (Fig. 8A-lower half, B).

### Biodegradability and non-biotoxic property of NS

Finally, we evaluated biodegradability and non-biotoxic property of NS. The course of degradation of NS over 1 year period was followed by 3D-CT. NS was implanted in the thigh muscle of normal mice. The main degradation mechanisms of PLLA in vivo are random hydrolytic chain scission, enzymatic degradation and acceleration of the breakdown by free radicals released from activated phagocytic cells such as macrophages [32]–[34]. Reduction in its molecular weight reduces the strength of NS, resulting in cracking and decomposition [32]. NS volume detected by 3D-CT decreased gradually over 1 year period, although 35.1±8.0% of NS volume was still detected 12 months after implantation (Fig. S1). Next, histological examination was performed to confirm the biodegradability and non-biotoxic property of NS. At 7 days, 3 months, and 12 months after implantation, a small number of CD45 positive leukocytes were recruited around NS, and most of these cells were Mac3 positive macrophages (Fig. S2). The macrophage count around NS was lesser at 7 days than at 3 and 12 months after implantation. At 12 months after implantation, invasion by macrophages inside the decomposed particles was noted (Fig. S2). These data suggested that the recruitment of macrophages around NS was due to the mild foreign-body reaction to eliminate foreign matter [35]. In addition, although slight increase in the number of fibroblast and the amount of type I and type III collagens around the particles were noted 12 months after implantation, no intense chronic inflammation, which could promote the strongly enhanced fibrogenesis by fibroblast [35], [36], was observed (Fig. S3).

## Discussion

In this study, we presented usefulness of a novel and feasible injectable cell scaffold to enhance cell-based therapeutic angiogenesis. Co-implantation of NS markedly prolonged the localization of implanted BMNCs in ischemic tissues (Fig. 2). Angiogenesis and collateral formation of arteriogenesis were significantly induced around the BMNC and NS clusters (Fig. 4, 5), resulting in significant limb salvage (Fig. 3). These results further emphasize that the prolonged localization of implanted cells is important for induction of effective neovascularization in ischemic tissues. Moreover, our nano-scaffold could be applied to various cell types, including autologous BMNCs, stem cells, EPCs, PBMNCs, and adipose-derived regenerative cells, for therapeutic angiogenesis [1]–[4], [6], [37], because HAp shows excellent adhesive properties to various types of cells [15].

Peptide hydrogel and collagen-based matrix have both been reported as injectable cell scaffolds in animal models [13], [38], [39]. These matrix scaffolds also provide an angiogenic environment for successfully promoting long-term cell survival and capillary formation. However, the cumbersome procedures and techniques to prepare matrix scaffold containing cells may be an obstacle to the treatment of large numbers of cells in clinical use. In addition, a large volume of matrix may be required to implant the same number of cells compared with simple cell implantation due to the limited cell retention capacity of the matrix scaffold (∼5×10^6^ cells/ml) [13], [38], [39]. This could limit the implantation of a sufficient number of cells for therapy, since the injection space in the myocardium and limb muscles is restricted. In contrast, the procedure for mixing our NS particles into cell solutions is much simpler, and the NS mixture barely affects the volume of the originally prepared cell solutions for implantation.

In the present study, NS markedly enhanced therapeutic angiogenesis and arteriogenesis, which showed good correlations with increased VEGF and FGF-2 levels in ischemic muscle. VEGF is a potent stimulator of endothelial cell mitogenesis and cell migration [3], [7]. FGF-2 is also crucial for arteriogenesis with efficient blood flow through the stabilisation of newly formed endothelial tubules by recruiting pericytes and smooth muscle cells [3], [7]. Our observations further suggest that the clusters containing NS, implanted cells, and recruited host cells may form a microenvironment containing secreted cytokines and growth factors, such as VEGF and FGF-2 (Fig. 7), which could also contribute to reduce apoptotic cell death [7], [11]. Transfection of donor cells with antiapoptotic transgenes, e.g., VEGF, FGF-2, Akt and Bcl-2, has been reported to enhance cell survival, resulting in augmentation of the proangiogenic cytokine production [6], [7]. Meanwhile, we for the first time demonstrated that NS-mediated prolonged localization of unmodified donor cells as a cluster in ischemic tissue could change the local environment, through accelerated expression of proangiogenic factors, improve cell survival and behaviour, and augment neovascularisation, without gene manipulation or artificial extracellular matrix.

The increase in tissue VEGF and FGF-2 levels shown in Fig. 6 does not appear to be as large as compared to the marked alleviation of apoptosis of transplanted BMNCs by NS (Fig. 8). Because tissue cytokine levels were measured using the tissue extracts from whole thigh muscle, the levels of these cytokines shown in Fig. 6 are the average of the entire ischemic muscle. Meanwhile, these cytokines were distributed in high concentrations in the area where the implanted cells and NS formed clusters (Fig. 7). Therefore, a significantly high cytokine level measured as the average for the entire muscle indicated that the effect of NS in amplifying the cytokine level in the implanted area was much larger than that shown in Fig. 6. Exposure to such high levels of cytokines in this area appears to markedly alleviate apoptosis (Fig. 8), and such a cytokine concentration gradient appears to be useful to induce efficient neovascularization in the implanted area [3].

Similar to VEGF and FGF-2, IL-1β, MCP-1, and SDF-1 are known as proangiogenic factors induced by ischemia [3], [4], [40]–[42]. In fact, increase in the expression of all proangiogenic factors was induced by ischemia alone 3 days after ischemic induction in the present study (Fig. 6, vehicle group). Although we observed additional increase in these 3 cytokines after cell implantation, the change observed was not significant enough to explain why marked improvements in blood flow and limb salvage were observed only in the NS+BMNCs group. Three days after transplantation/ischemic induction, IL-1β and MCP-1 levels tended to be high in the NS+BMNCs group, though there was no significant difference in the levels between the NS+BMNCs group and the LA+BMNCs group. SDF-1 expression was not affected by cell implantation after 3 days. Seven days after transplantation/ischemic induction, tissue IL-1β, MCP-1, and SDF-1 levels were significantly higher in the cell-implanted groups (BMNCs group, LA+BMNCs group, and NS+BMNCs group) than in the vehicle group and the NS alone group (Fig. 6). This is probably because large amounts of VEGF and FGF-2 are mainly secreted from implanted cells [3], [7], [8], [43], while IL-1β, MCP-1, and SDF-1 are additionally secreted from muscle cells, endothelial cells, and stromal cells activated by the implanted cells [41], [43]–[46]. In the present study, neovascularization was not stimulated by the elevation of these cytokines 7 days after implantation in the BMNCs alone group and the LA+BMNCs group. The exact reason for this finding is unknown. One possible explanation is the interaction with other proangiogenic factors. For neovascularization, coordinated actions of multiple proangiogenic factors appear to be important [3], [47]. It appears that in the BMNCs alone group and the LA+BMNCs group, these cytokines failed to function satisfactorily because VFGF levels decreased to that of the non-ischemic group (sham) 7 days after ischemic induction (Fig. 6-a). VEGF has been reported to play an important role for SDF-1 activity [47], which might also be true for IL-1β and MCP-1.

There are still several limitations to the use of NS for cell-based therapeutic angiogenesis in clinical and practical use. First, it is necessary to control the absorption time of NS in the ischemic tissues to ensure both therapeutic efficacy and safety. When normal mice were followed by 3D-CT for 1 year after NS implantation, NS decreased gradually over time, but 35.1% of the particle volume remained after 1 year (Fig. S1). Although too rapid absorption can reduce the cell-retaining potential, it appears that modulation of NS is necessary to accelerate its absorption appropriately to avoid residual foreign particle accumulation in the living body. To achieve appropriate absorption, other biodegradable polymers (*e.g.*, polyglycolic acid (PGA), polycaprolactone (PCA) or copolymers, such as poly(lactic-*co*-glycolic acid) (PLGA)) could be alternate substitutes [48], [49].

Second, tissue toxicity of biodegradable polymers needs to be taken into account. Although the formation of collaterals did not differ between the BMNCs group and the LA+BMNCs group (Fig. 5B), limb survival significantly improved in the BMNCs alone group but not in the LA+BMNCs group (Fig. 3). Hence, tissue toxicity by LA cannot be ruled out. PLLA has already been applied clinically as a safe biodegradable material [21], [50]. However, several reports suggest that the substances formed by degradation of PLLA may have adverse effects on tissue [32], [51]. The lack of improvement of limb survival in the LA+BMNCs group may be attributable to such adverse effects. In contrast, NS appears to have no tissue toxicity. When NSs were implanted in the thigh muscle of normal mice, a small number of macrophages were observed around NSs, and there was no severe inflammatory reaction in the implanted area (Fig. S2). Further, at 12 months after implantation, enhanced fibrillization around the NS particles was not observed (Fig. S3). Thus, NS, but not LA, appears to have no tissue toxicity, in agreement with previous our reports [18], [20], [52]. One possible reason for this result is that the HAp nanoparticles covering the NS surface probably serve as a buffer agent, preventing pH reduction arising from PLLA degradation [18], [20], [53]. The formation of lactic acid and its oligomer from PLLA degradation reduces pH, and this change is reported to cause inflammatory reactions [32], [51]. The product from dissolution of HAp nanocrystals (basic calcium phosphate) has been suggested to serve as a buffer for such reactions [53]. Thus, it appears that coating of NS with HAp nanoparticles resulted not only in cell adhesiveness but also increased safety. However, to ensure further safety, selection of appropriate biodegradable polymers is essential.

Third, it is also necessary to estimate the appropriate procedures for treatment, *i.e.*, the appropriate ratio of cells to NS and the preincubation time of cells and NS prior to implantation. Regarding preincubation time, our preliminary data suggested that efficacy of cell-based therapeutic angiogenesis was not diminished even when the step of preincubation of cells with NS was omitted. This result makes this procedure much simpler for clinical use. Meanwhile, there might be scope for improving the conditions for preincubation of cells and NS. For example, additional therapeutic efficacy may be achieved if hypoxic preconditioning [54] is used in combination with the current preincubation conditions. It is reported that preincubation of progenitor cells under hypoxic condition induces the expression of SDF-1 receptor, CXCR4, increasing the benefit of progenitor cell therapy [54]. SDF-1 plays a critical role in recruitment, tissue retention, and cell survival of hematopoietic cells and bone marrow-derived progenitor cells, which promote angiogenesis and tissue regeneration [42]. CXCR4 is normally expressed in hematopoietic cells and progenitor cells, and is upregulated by hypoxia [42], [55], [56]. As shown in Fig. 6, high levels of SDF-1 expression are observed in ischemic tissue [42]. Hence, increasing the expression of CXCR4 in implanted cells under hypoxic condition through preincubation of NS and cells may further improve tissue retention and cell survival after cell implantation mediated by the SDF-1/CXCR4 axis.

Fourth, care may be needed to prevent embolism due to particles at the time of implantation. However, this event can be easily avoided because excessive injection is unlikely to occur unless direct injection into blood vessels occurs carelessly during intramuscular injection. Even with slight injection into blood vessels, the influence of minor microembolism arising from 100 µm diameter particles will be negligibly small.

In conclusion, we have developed a novel type of injectable cell scaffold and, for the first time, demonstrated that it is a simple and easy device for use as an enhancer of cell-based therapeutic angiogenesis. This nano-scaffold provides promising local environment for implanted cells for the effects on angiogenesis and arteriogenesis through cell clustering, augmented expression of proangiogenic factors, and supporting cell survival without gene manipulation or artificial extracellular matrix.

## Supporting Information

Figure S1 **The course of degradation of NS followed by 3D-CT.** (A) NS (30,000 particles) was implanted in the thigh muscle on one side of each normal mouse (C57BL/6NCrSlc). The course of degradation of NS over 1 year period was followed by 3D-CT of live mice immediately and 6 months and 12 months after implantation. Representative 3D-CT images obtained from the same mouse at each point of time are shown. PLLA microspheres containing magnetite (PLA-Particles-M) are used as the core of NS to be detected by X-ray 3D-CT. The NS detected by 3D-CT were visualised as green particles. (B) Quantitative volume analysis of NSs was performed using 3D-CT data obtained from 3 mice immediately and 6 months and 12 months after implantation. Residual NS volume was expressed as a percentage of the NS volume immediately after implantation in each mouse. Data are shown as means (SD). Abbreviations: NS, nano-scaffolds; 3D-CT, three-dimensional computed tomography.
(TIF)

Figure S2 **Inflammatory reactions around NS.** NS (3,000 particles) was implanted in the thigh muscle on one side of each normal mouse (C57BL/6NCrSlc). Tissues around NS were examined 7 days, 3 months and 12 months after implantation for inflammatory reactions. Tissue sections were counterstained with DAPI (blue), and immunofluorescence was detected by staining with anti-CD45 antibody (red) and anti-Mac3 antibody (green). Scale bars: 100 µm. Abbreviations: NS, nano-scaffolds.
(TIF)

Figure S3 **Collagen accumulation around NS 12 months after implantation.** NS (3,000 particles) was implanted in the thigh muscle on one side of each normal mouse (C57BL/6NCrSlc). Tissues around NS were examined 12 months after implantation for enhanced fibrillization. Tissue sections were counterstained with DAPI (blue), and immunofluorescence was observed with anti-fibroblast antibody (red) and anti-collagen I antibody (green), or anti- collagen III antibody (green). The upper panels of each group show normal muscle tissue (normal) and the lower panels show the area around NS (NS). Arrowheads indicate slightly accumulated type I collagen, and arrows indicate slightly accumulated type III collagen. Scale bars: 100 µm. Abbreviations: NS, nano-scaffolds.
(TIF)

Methods S1 **Supplemental methods.**
(DOCX)
