# Supplementary figures and images for: Enhancement of Cell-Based Therapeutic Angiogenesis Using a Novel Type of Injectable Scaffolds of Hydroxyapatite-Polymer Nanocomposite Microspheres
Source: PLoS One. 2012 Apr 18;7(4):e35199. doi: 10.1371/journal.pone.0035199 (PMC3329450; doi:10.1371/journal.pone.0035199)

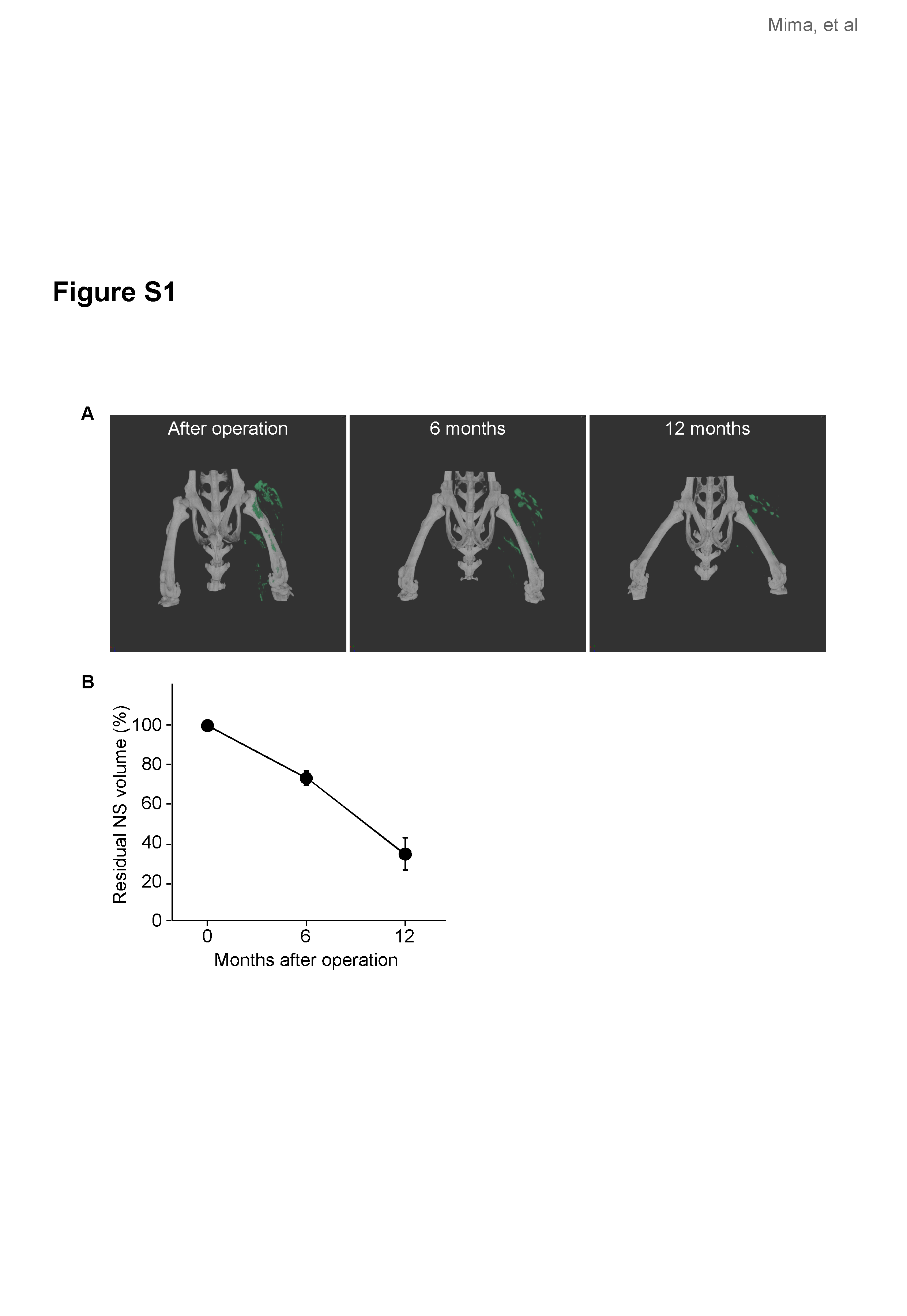

Supplement: Figure S1 — The course of degradation of NS followed by 3D-CT. (A) NS (30,000 particles) was implanted in the thigh muscle on one side of each normal mouse (C57BL/6NCrSlc). The course of degradation of NS over 1 year period was followed by 3D-CT of live mice immediately and 6 months and 12 months after implantation. Representative 3D-CT images obtained from the same mouse at each point of time are shown. PLLA microspheres containing magnetite (PLA-Particles-M) are used as the core of NS to be detected by X-ray 3D-CT. The NS detected by 3D-CT were visualised as green particles. (B) Quantitative volume analysis of NSs was performed using 3D-CT data obtained from 3 mice immediately and 6 months and 12 months after implantation. Residual NS volume was expressed as a percentage of the NS volume immediately after implantation in each mouse. Data are shown as means (SD). Abbreviations: NS, nano-scaffolds; 3D-CT, three-dimensional computed tomography. (TIF) [file pone.0035199.s001.tif]

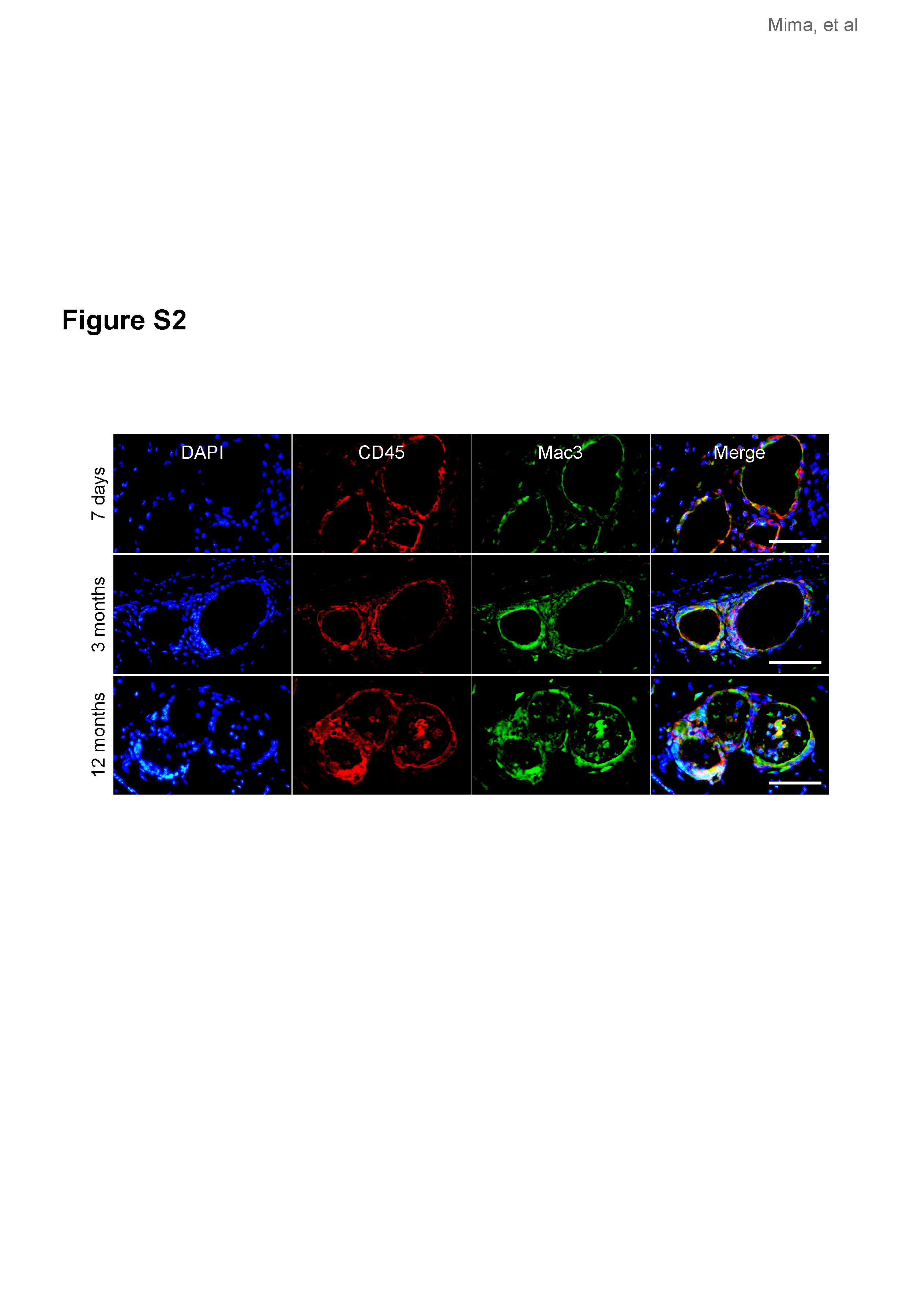

Supplement: Figure S2 — Inflammatory reactions around NS. NS (3,000 particles) was implanted in the thigh muscle on one side of each normal mouse (C57BL/6NCrSlc). Tissues around NS were examined 7 days, 3 months and 12 months after implantation for inflammatory reactions. Tissue sections were counterstained with DAPI (blue), and immunofluorescence was detected by staining with anti-CD45 antibody (red) and anti-Mac3 antibody (green). Scale bars: 100 µm. Abbreviations: NS, nano-scaffolds. (TIF) [file pone.0035199.s002.tif]

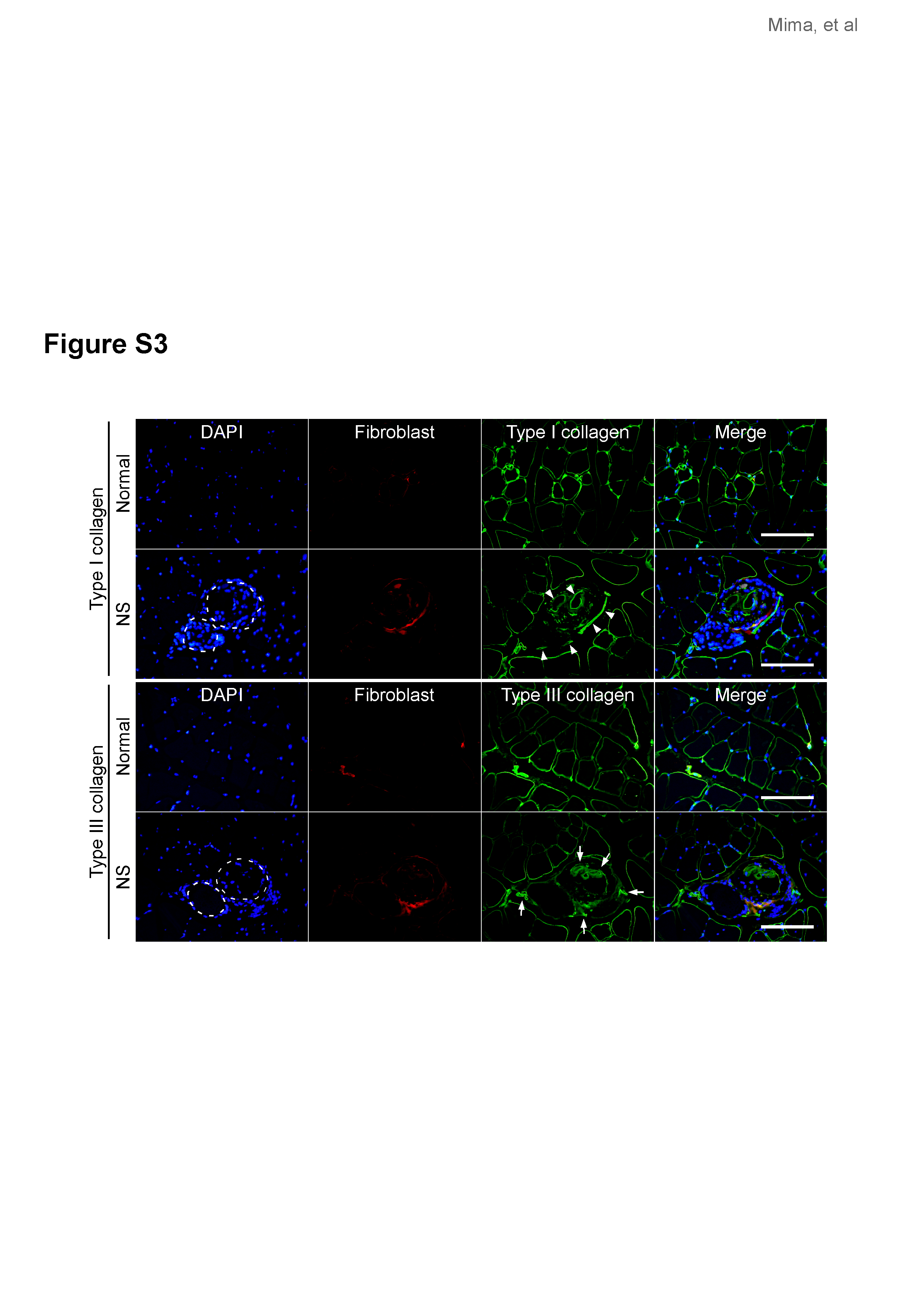

Supplement: Figure S3 — Collagen accumulation around NS 12 months after implantation. NS (3,000 particles) was implanted in the thigh muscle on one side of each normal mouse (C57BL/6NCrSlc). Tissues around NS were examined 12 months after implantation for enhanced fibrillization. Tissue sections were counterstained with DAPI (blue), and immunofluorescence was observed with anti-fibroblast antibody (red) and anti-collagen I antibody (green), or anti- collagen III antibody (green). The upper panels of each group show normal muscle tissue (normal) and the lower panels show the area around NS (NS). Arrowheads indicate slightly accumulated type I collagen, and arrows indicate slightly accumulated type III collagen. Scale bars: 100 µm. Abbreviations: NS, nano-scaffolds. (TIF) [file pone.0035199.s003.tif]
