# Supplementary material for: Enhancement of Cell-Based Therapeutic Angiogenesis Using a Novel Type of Injectable Scaffolds of Hydroxyapatite-Polymer Nanocomposite Microspheres
Source: PLoS One. 2012 Apr 18;7(4):e35199. doi: 10.1371/journal.pone.0035199 (PMC3329450; doi:10.1371/journal.pone.0035199)
Supplement: Methods S1 — Supplemental methods. (DOCX) [file pone.0035199.s004.docx]

**Supplemental Methods:**

**Enhancement of Cell-Based Therapeutic Angiogenesis using a Novel Type of Injectable Scaffolds of Hydroxyapatite-Polymer Nanocomposite Microspheres**

Yohei Mima, Shinya Fukumoto, Hidenori Koyama, Masahiro Okada, Shinji Tanaka, Tetsuo Shoji, Masanori Emoto, Tsutomu Furuzono, Yoshiki Nishizawa, Masaaki Inaba

**Inventory of Supplementary information**

Supplementary experimental procedures

Materials

3D-CT analysis and volume measurement of implanted NSs

Immunohistochemistry of inflammatory cells, fibroblasts and collagens

**Supplementary experimental procedures**

**Materials**

The PLLA microspheres containing magnetite (PLA-Particles-M) were purchased from Micromod Partikeltechnologie (Rostock, Germany). Rat anti-CD45 antibody (clone: 30-F11) was obtained from BD Pharmingen (San Diego, CA). Rabbit anti-Mac3 antibody was obtained from AbD Serotec (Oxford, UK). Rat anti-Fibroblast antibody (clone: ER-TR7) was purchased from BMA Biomedicals AG (Augst, Switzerland). Rabbit anti-Collagen I antibody and rabbit anti-Collagen III antibody were obtained from Abcam (Cambridge, UK). Alexa Fluor 594-conjugated goat anti-rat IgG antibody and Alexa Fluor 488-conjugated goat anti-rabbit IgG antibody were purchased from Invitrogen Life Technologies (Carlsbad, CA). 4',6-Diamino-2-phenylindole dihydrochloride (DAPI) was obtained from Pierce Biotechnology (Rockford, IL).

**3D-CT analysis and volume measurement of implanted NSs**

We used PLLA microspheres containing magnetite (PLA-Particles-M) as the core of NS to be detected by X-ray CT scan. C57BL/6NCrSlc mice were anaesthetised with sodium pentobarbital (50 μg/g, i.p.), and CT images were obtained using a micro-CT scanner (La Theta LCT200; Aloka, Tokyo, Japan) according to the manufacturer’s protocol. The 3D data were constructed from sliced CT images by summing those images along the Z-axis, and NS volume analysis was performed with an image analyzer (VGStudio MAX software; Volume Graphics, Heidelberg, Germany) according to the manufacturer’s protocol.

**Immunohistochemistry of inflammatory cells, fibroblasts and collagens**

C57BL/6NCrSlc mice were anaesthetized with pentobarbital (i.p.) and perfused through the left ventricle using 20 ml of saline followed by 100 ml of cold 4% paraformaldehyde (PFA) in phosphate buffer (PB). The thigh tissue was post-fixed in 4% PFA for 2 h. The tissue was then embedded in paraffin or immersed in 20% sucrose in PB overnight followed by freezing in OCT compound (Sakura Finetek, Co. Japan). The frozen sections were incubated with anti-CD45 antibody and anti-Mac3 antibody, or anti-Fibroblast antibody and anti-Collagen I antibody or anti-Collagen III antibody, followed by incubation with fluorescent secondary antibodies and counterstaining with DAPI. Immunofluorescence was observed using a fluorescence microscope (BZ-8000; Keyence, Osaka, Japan).
